# The rise of digital direct-to-consumer advertising?: Comparison of direct-to-consumer advertising expenditure trends from publicly available data sources and global policy implications

**DOI:** 10.1186/s12913-015-0885-1

**Published:** 2015-06-19

**Authors:** Tim K. Mackey, Raphael E. Cuomo, Bryan A. Liang

**Affiliations:** Department of Anesthesiology, University of California, San Diego School of Medicine, San Diego, CA USA; Division of Global Public Health, Department of Medicine, University of California, San Diego School of Medicine, San Diego, CA USA; Global Health Policy Institute, University of California, San Diego, CA USA; Joint Doctoral Program in Global Public Health, University of California, San Diego – San Diego State University, San Diego, CA USA

**Keywords:** Direct-to-consumer advertising, Sunshine act, Physician payments, Conflicts of interest, Health marketing and promotion, Health policy, eHealth

## Abstract

**Background:**

Pharmaceutical marketing is undergoing a major shift in the United States, in part due to new transparency regulations under the healthcare reform act. Changes in pharmaceutical marketing practices include a possible shift from more traditional forms of direct-to-consumer advertising towards emerging use of Internet-based DTCA (“eDTCA”) given the growing importance of digital health or “eHealth.” Though legally allowed only in the U.S. and New Zealand, eDTCA poses novel regulatory challenges, as it can cross geopolitical boundaries and impact health systems and populations outside of these countries.

**Methods:**

We wished to assess whether changes in DTCA and eDTCA expenditure trends was occurring using publicly available pharmaceutical marketing data. DTCA data was analyzed to compare trends in aggregate marketing expenditures and to assess if there were statistically significant differences in trends and magnitudes for data sources and DTCA sub-categories (including eDTCA). This was accomplished using regression lines of DTCA trend data and conducting pairwise comparisons of regression coefficients using t-tests. Means testing was utilized for comparing magnitude of DTCA expenditure.

**Results:**

Data from multiple data sources indicate that aggregate DTCA expenditures have slightly declined during the period from 2005–2009 and are consistent with results from other studies. For DTCA sub-categories, television remained the most utilized form of DTCA, though experienced trends of declining expenditures (−13.2 %) similar to other traditional media platforms such as radio (−30.7 %) and outdoor ads (−12.1 %). The only DTCA sub-category that experienced substantial increased expenditures was eDTCA (+109.0 %) and it was the only medium that had statistically significant differences in its marketing expenditure trends compared to other DTCA sub-categories.

**Conclusions:**

Our study indicates that traditional DTCA marketing may be on the decline. Conversely, the only DTCA sub-category that experienced significant increases was eDTCA. However, to fully understand this possible shift to “digital” DTCA, improvements in publicly available DTCA data sources are necessary to confirm changing trends and validate existing data. Hence, utilizing the newly implemented U.S. physician-payment expenditure transparency requirements, we advocate for the mandatory disclosure of DTCA/eDTCA in order to inform future domestic and international health policy efforts regarding appropriate regulation of pharmaceutical promotion.

## Background

Pharmaceutical marketing is currently undergoing a unique and understudied transition in the United States, the world’s largest market for prescription drug sales [1]. In the 1990s, expanding pharmaceutical industry marketing budgets aggressively targeted physicians through deployment of tens of thousands of pharmaceutical sales representatives and use of other promotional strategies [2, 3]. During this same time period, prescription drug spending experienced a nearly six-fold increase from $40.3–$234.1 billion between 1990–2008 [4, 5]. The rapid rise in pharmaceutical marketing activities was also accompanied by the emergence of a relatively new and previously underutilized form of pharmaceutical promotion, but one directed at the consumer, not the physician [6, 7]. The emergence of direct-to-consumer advertising (“DTCA”) in the late 1990s - a form of pharmaceutical marketing that directly advertises prescription drugs and targets the consumer/patient – represented a new opportunity for industry marketing diversification and influencing prescription drug sales and utilization [6–8]. Yet, this marketing phenomenon is unique, as it is only permitted in the U.S. and New Zealand among developed countries, though forms of direct and indirect promotion to consumers (e.g., promotional materials, reminder advertisements, “infomercials,” and unbranded advertising campaigns) can occur outside these two countries [6, 9–12].

Similar to forms of physician-directed promotion, DTCA has been criticized as leading to inappropriate prescribing (initiated by patients, not prescribers), overemphasis of benefits versus risks in marketing claims, and overutilization of prescription and branded drugs [13–18]. Due to these concerns, criticism has been leveled against DTCA and calls for limits have been explored, but have failed to gain traction largely due to constitutional challenges in the U.S. on restricting forms of commercial free speech [6, 13, 16]. Indeed, calls for an outright ban on DTCA mimic the 25-year old WHO Ethical Criteria for Medicinal Drug Promotion that first recommended the general prohibition of DTCA even before its popularization and widespread use in the U.S. [19]. Additionally, DTCA has also gone through its own changes as the regulatory and pharmaceutical market environment around it has developed, including rapid expansion of use following liberalization of US Food and Drug Administration (“FDA”) regulation/guidance in the 1980s and late 1990s that lead to an estimated ~330 % increase in expenditures from 1996–2005, but also experiencing recent declines in expenditures following the global economic crisis and patent expiration of a number of blockbuster drug products [7, 12, 13, 20–22].

At the same time, a new era of digital health information-seeking and behavior has given rise to new sub-categories of DTCA - including DTCA that is Internet-based or uses social media technology/platforms (“eDTCA”) - that have not been well quantified or studied [9, 10, 23, 24]. Despite eDTCA’s potential to emerge as a more predominant health-marketing medium in this new era of the “e-patient,” there remains a general lack of transparency of data reporting *both* DTCA and eDTCA expenditures that is needed to assess emerging trends and fundamental changes in health marketing strategies. Hence, we wished to examine whether shifts in DTCA and eDTCA expenditures were actually occurring. We also wanted to assess whether there was adequate access and availability to data reporting DTCA expenditures in order to explore this question. Finally, we explore the potential health policy implications and necessary solutions to address the changing nature of DTCA.

## Methods

We first set out to identify publicly available data sources that collect information about DTCA and DTCA sub-category (including eDTCA) expenditures. Such identification was consistent with methods previously reported in the literature [13, 20]. Specifically, they include utilizing data from the following marketing firms: IMS Health, Nielsen Co., Cegedim Strategic Data, and Kantar Media (collectively “Marketing Firms”). Data utilized for this study were either free or required a fee to purchase (but were nevertheless accessible to the general public and did not require a formal data request or proposal for access.) We excluded data sources that required a formal research proposal for licensing, institutional subscription fees, and/or fee-based academic/research licenses generally not offered to the public. We focused on data in the public domain as we wished to assess the level of transparency of DTCA expenditure information and whether such information was readily available for public scrutiny absent the payment of oftentimes expensive fees for licensing the data (commonly paid for by industry for market research purposes) or submission of a proposal providing rationale to access/license the data (which can be time intensive, subject to certain use restrictions or pre-publication review, and which could be rejected by a Marketing Firm.) Though we did not seek data directly from Marketing Firms, we note that a request for DTCA data and DTCA sub-categories (such as eDTCA) generally requires a fee-based license and formal data request proposal directly with Marketing Firms. As an example, IMS Health has a lengthy process for licensing data, which includes submitting a detailed request, concept assessment, finalizing project specifications, and finally contracting, payment, and delivery. Overall, these requirements may make it difficult or cost prohibitive to obtain DTCA expenditure data.

The majority of publicly available DTCA data examined was gathered through trade publications and/or other formats (e.g. press releases, white papers, and third-party licensee fee-based reports). All data sources assessed in this study have been listed in Appendix Table 1. Data collection methods utilized by these sources were predominantly conducted using surveys of national media outlets (including TV, radio, print, Internet, etc.) and other media channels. In addition to Marketing Firm non-peer reviewed reports, we also utilized a recent publication by *Kornfield et al.* in the journal PLoS One for comparison purposes as it is the most comprehensive and up-to-date source on aggregate pharmaceutical promotion expenditure [20]. *Kornfield et al.* utilized Kantar Media and IMS Health as data sources in their analyses. We examined data from 2005–2009 in our DTCA expenditure analysis, as it was the only time period where DTCA data were available for aggregate spending and for DTCA sub-categories examined/compared when this study was conducted.

**Table 1:** Percentage change in categories of US DTCA expenditures (2005–2009)

DTCA category	2005 expenditure/rank	2009 expenditure/rank	Percentage change (2005–2009)
Total television^a^	$3,390,587,472 (1)	$2,943,000,894 (−)	−13.20 %
Total print media^b^	$1,320,786,065 (2)	$1,349,517,112 (−)	2.18 %
Total radio outlets^c^	$66,798,340 (3)	$46,323,067 (4)	−30.65 %
Outdoor ads	$8,640,720 (5)	$7,598,381 (−)	−12.06 %
Internet	$56,180,283 (4)	$117,403,346 (3)	108.98 %
Total DTCA expenditure	$4,842,992,880	$4,463,842,800	−7.83 %

Source: Nielsen Co. data sourced from fee-based PharmLive Report “Direct-to-Consumer Advertising: Review and Outlook 2011”

^a^Includes network, cable, syndicated, and spot TV ads. Excludes Spanish Language TV DTCA expenditures

^b^Includes national magazine, local magazine, national Sunday supplement, local Sunday supplement, national newspaper, and local newspaper

^c^Includes network and spot radio ads

Data were analyzed to assess trends (both increasing and decreasing) in DTCA marketing expenditures and to assess if there were statistically significant differences in trends and magnitudes for data sources and DTCA sub-categories. In order to assess overall expenditure change for DTCA sub-categories over the five-year period from 2005–2009, percentage change calculations were conducted (Table 1). In order to assess statistically significant differences in trends, regression lines were computed for the series of years available from each data source and each data category. Pairwise comparisons of regression coefficients were then conducted using t-tests (Tables 2 and 3). In order to assess statistically significant differences in magnitude, means were computed for the series of years available from each data source and each data category. These means were then adjusted by estimating a common slope. t-Tests were then used to determine if differences in magnitude were statistically significant.

**Table 2:** Pairwise statistical tests to determine (1) if trendline slopes for 2005–2009 DTCA data types were significantly different and (2) if magnitudes for 2005–2009 DTCA data types were significantly different after adjusting for effect of time

	Statistic	TV vs. Print	TV vs. Radio	TV vs. Outdoor	TV vs. Internet
Trend	Δ (2nd minus 1st)	-$122,609,071	-$146,138,808	-$154,272,982	-$167,279,709
	SE_diff_	$94,728,227	$64,642,885	$64,409,125	$64,550,379
	t	−1.2943	−2.2607	−2.3952	−2.5915
	df	6	6	6	6
	*p*	0.2431	0.0645	0.0536	0.0411
Magnitude	Δ (2nd minus 1st)	-$1,706,354,882	-$3,137,268,387	-$3,177,916,521	-$3,099,469,979
	SE_diff_	$140,278,912	$115,175,353	$117,948,373	$123,036,591
	t	−12.164	−27.2391	−26.9433	−25.1914
	df	7	7	7	7
	*p*	<0.0001	<0.0001	<0.0001	<0.0001
Print vs. Radio	Print vs. Outdoor	Print vs. Internet	Radio vs. Outdoor	Radio vs. Internet	Outdoor vs. Internet
$23,529,737	-$31,663,911	-$44,670,638	-$8,134,174	-$21,140,901	-$13,006,727
$69,692,386	$69,475,618	$69,606,591	$5,668,070	$7,095,286	$4,491,759
0.3376	−0.4558	−0.6418	−1.4351	−2.9796	−2.8957
6	6	6	6	6	6
0.7471	0.6646	0.5448	0.2013	0.0247	0.0275
$1,430,913,505	-$1,471,561,639	-$1,393,115,097	-$40,648,133	$37,798,408	$78,446,542
$92,111,455	$92,526,092	$94,212,421	$8,601,111	$14,628,702	$9,106,204
15.5346	−15.9043	−14.787	−4.7259	2.5839	8.6146
7	7	7	7	7	7
<0.0001	<0.0001	<0.0001	0.0021	0.0363	<0.0001

Note: TV expenditures reported exclude Spanish TV DTCA

**Table 3:** Pairwise statistical tests to determine (1) if trendline slopes for 2005–2009 Internet DTCA data sources were significantly different and (2) if magnitudes for 2005–2009 Internet DTCA data sources were significantly different after adjusting for effect of time

	Statistic	Nielson vs. Kantar
Trend	Δ (2nd minus 1st)	-$5,348,460
	SE_diff_	$28,107,994
	t	−0.1903
	df	6
	*p*	0.8554
Magnitude	Δ (2nd minus 1st)	$107,111,591
	SE_diff_	$36,912,878
	t	2.9017
	df	7
	*p*	0.0229

## Results

**Fig. 1:**
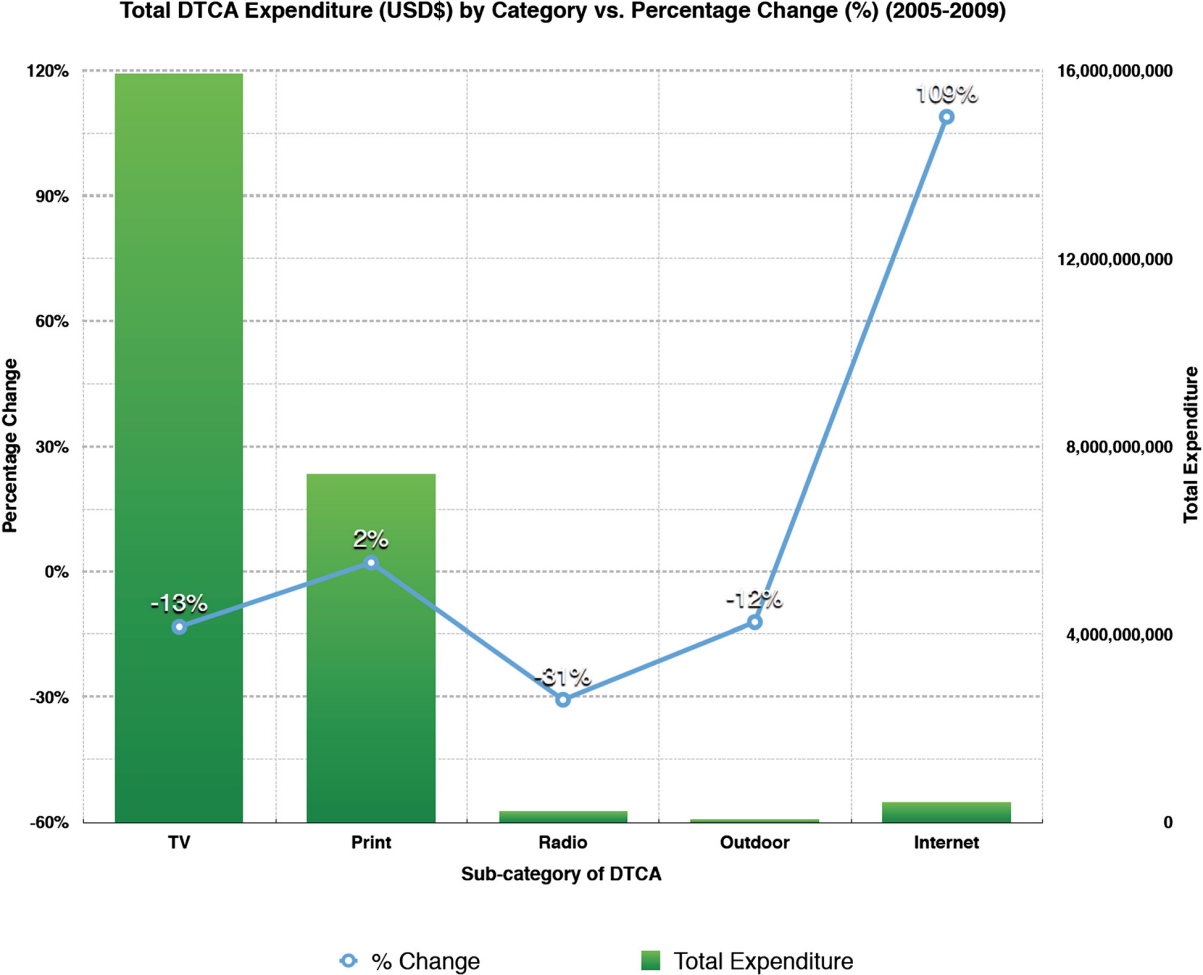
Total DTCA Expenditure (USD$) by Category vs. Percentage Change (%) (2005-2009)

Information from multiple data sources indicate that aggregate DTCA expenditures have slightly declined (−7.83 %) during the period from 2005–2009. Additionally, Nielsen Co. DTCA expenditure data was examined for an additional five sub-categories of DTCA: television, print media, radio, outdoor marketing, and specifically Internet (i.e. eDTCA) with differences reported in total expenditure and percentage increase from 2005–2009 (Fig. 1). We excluded in our analysis DTCA sub-categories of Spanish language TV and free standing insert coupons as this data was not consistently collected during the time period examined from data sources.

Television remained the largest sub-category of DTCA expenditure in both 2005 and 2009, although it exhibited a 13.20 % decline over that time period. Print media was the second-largest type of DTCA expenditure in both 2005 and 2009; its expenditures remained approximately the same, exhibiting only a 2.18 % increase and it did not significantly differ from trends in any other types of DTCA expenditure. Radio expenditure was the third-largest type of DTCA expenditure in 2005 and the fourth-largest type of DTCA expenditure in 2009, exhibiting a 30.65 % decline. Outdoor marketing expenditure was the smallest type of DTCA expenditure in both 2005 and 2009, exhibiting a 12.06 % decrease (Table 2).

**Fig. 2:**
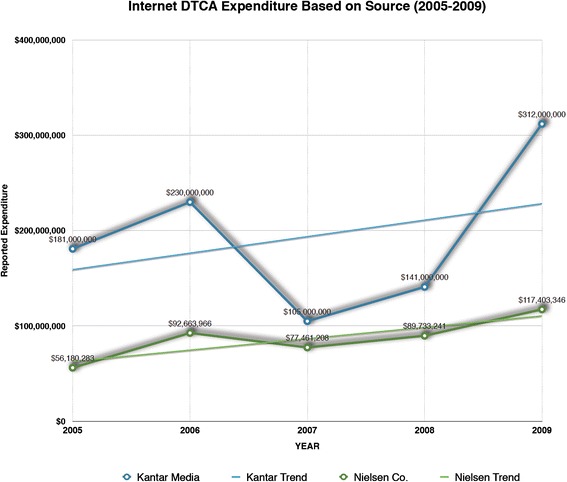
Internet DTCA Expenditure Based on Source (2005-2009)

Internet expenditure was the fourth-largest type of DTCA expenditure in 2005 and the third-largest type of DTCA expenditure in 2009. It was also the only DTCA sub-category to experience a triple-digit increase (108.98 %) in expenditure (with print DTCA only experiencing a modest 2.18 % increase over the same time period). Its upward trend was the only expenditure data significantly different from the downward trends of expenditures in television (p = 0.0411), radio (p = 0.0247), and outdoor (p = 0.0275) types of DTCA (Table 2). Kantar Media also reported upward trends for Internet DTCA expenditure. Though all sources reported an upward trend in eDTCA, Kantar Media reported a significantly higher amount (+$107,111,591) of Internet DTCA expenditure than Nielsen Co. (p = 0.0229) (See Fig. 2 and Table 3).

From a data source comparison perspective, Nielsen Co. did not report a significantly different magnitude of total DTCA expenditure than Kantar Media (p = 0.5144). Furthermore, significant differences in magnitude did not exist between IMS Health and Nielsen Co. (p = 0.3145), IMS Health and Kantar Media (p = 0.0971), Nielsen Co. and data from the *Kornfield et al.* PLoS One study (p = 0.073), and Kantar Media and PLoS One (p = 0.2495). The only significant difference in reporting of total DTCA expenditure data sources was between IMS Health and PLoS One (p = 0.017), in which IMS Health reported a $620,666,667 lower magnitude of DTCA expenditure than the PLoS One study. All data sources reported that DTCA expenditures first increased and then decreased over the time period reviewed and no significant difference in total, overall DTCA expenditure trends was detected.

## Discussion

Our examination indicates that the expenditure trend in traditional forms of DTCA (TV, print, radio) is statistically significantly different than that for eDTCA. Specifically, while traditional media types of DTCA expenditure has been diminishing, eDTCA expenditure has experienced three-digit percentage growth, though admittedly remains much lower in absolute spending compared to other historically dominant sub-categories of DTCA (primarily TV broadcast). However, given the popularity and increasing use of the Internet to search for and consume health information, it is not unreasonable to expect that increases in eDTCA expenditures will continue, though without reliable data, the growth and impact may not fully be understood [9, 10, 23].

Further, some observed lack of agreement across different data sources for DTCA expenditure indicates that there is a general absence of consistent, accessible, and high-quality data needed for future health policy and regulatory decision-making when assessing the changing use and impact of DTCA [1]. Despite a general alignment of aggregate DTCA expenditure and the trend directionality, lack of robust disclosure of DTCA expenditure and the sampling methodology used to collect such data makes comparisons difficult to validate. Despite these limitations, we nevertheless were able to identify that declining trends in aggregate DTCA spending contrasted with a rise of Internet-based DTCA, though variation in data sources makes it difficult to know the exact magnitude.

While declines in aggregate DTCA are largely unsurprising, given recent challenges faced by the pharmaceutical industry and the US economy during the global financial crisis that led to declines in overall marketing budgets [20], our findings showing an increase in eDTCA expenditures seem to run contrary to these general market trends. Specifically, even during financially straining periods, increases in eDTCA may indicate that marketers are simply retargeting their promotion to where consumers increasingly seek and consume health information and products: online. Indeed, reflecting this potential trend, the Pew Internet Research estimates 72 % of adult users now actively search for health information online [23]. In addition, US healthcare and pharmaceutical online advertisement spending was predicted to experience double-digit percent growth from 2010–2015 [10]. Hence, our findings may simply reflect a trend of increasing utilization of the Internet by health consumers and its resultant effect of shifting drug advertising away from traditional forms of DTCA to eDTCA that may also be cheaper and more accessible for marketers [1, 25].

Most importantly, increases in US-based eDTCA expenditure may also pose unique global public policy concerns that have yet to be adequately assessed. Specifically, eDTCA has already been identified as easily transmitting over geopolitical borders via satellite TV, the Internet, and social media, consequentially becoming “globalized” given that it is disseminated and can be easily accessed by consumers worldwide [10, 25, 26]. Lack of access to robust data to make meaningful comparisons between DTCA and eDTCA expenditure trends further inhibits any ability to appropriately assess the international spread of eDTCA and its impact on populations outside of the U.S. and New Zealand, the only two developed markets where it is legally permissible [6, 27, 28]. This could lead to negative impacts on public health outcomes and national prescription drug expenditures in countries where such promotion is legally prohibited but not contained nor identified as a possible public policy problem [6, 10]. Additionally, existing regulatory frameworks addressing traditional DTCA may be ineffectual when responding to the rapid advances of Internet-based technologies and the dynamic engagement and interaction elements of social media based eDTCA [9, 10]. Specifically, the development of regulations and technical tools needed to legally limit dissemination within the U.S. market have not kept pace up with the rise of new eDTCA platforms, offerings, and various channels of consumer engagement [9].

Policymaking challenges include ongoing efforts by the FDA, which is struggling to formulate a regulatory framework to address social media-based DTCA. Steps taken by the FDA include issuing draft guidance documents emphasizing the need for communications in advertising to be balanced, accurate and non-misleading; requiring manufacturers to disclose benefit and risk information even when limited character space is available; how to respond to requests for off-label information; and addressing how firms should correct misinformation about their products posted by third-parties [29]. While still in draft form, several pharmaceutical firms and trade groups (including PhRMA and BIO), have been highly critical of FDA’s approach in this space, with finalization of FDA guidance still in progress following completion of a public comment period [30].

In addition, lack of sufficient eDTCA regulation can also enable clearly illicit online marketing that harms patients [31]. An example is the growth of illicit online pharmacies, which use their own forms of fraudulent and misleading eDTCA to illegally sell pharmaceutical products without prescriptions directly to the consumer [31–35]. These products may in fact be “falsified” or “fraudulent” (defined as medicines made with criminal intent to deceive regarding the authenticity or origin), as warned by the World Health Organization (“WHO”) and various drug regulatory agencies [9, 25, 36]. Consequentially, consumers may have trouble differentiating between eDTCA originating from fraudulent online pharmacies and eDTCA from authorized manufacturers, thereby leading to potential patient safety risks [31]. Organizations including the WHO, FDA, the UN Office of Drugs and Crime, and Interpol have begun to recognize the need for action against this dangerous form of illicit global e-commerce, but have yet to target eDTCA as a primary enabling factor and marketing medium for consumer access [31]. This may in part be due to the paucity of consistent data comparing legitimate and illegal forms of eDTCA for policy development.

Hence, one crucial step in appropriately assessing the potential health impacts of DTCA and its changing sub-categories is requiring disclosure and transparency similar to legal mandates for other forms of pharmaceutical marketing [1, 31]. Taking lessons from the recent passage of US Federal legislation under the Affordable Care Act that now requires transparency and annual public disclosure of certain marketing directed to physicians by drug, biologic and medical device manufacturers (also known as the “Sunshine” or transparency act provisions and OPEN PAYMENTS system), requiring mandatory disclosure of DTCA and *specifically* of its sub-category of eDTCA, would provide important information in determining accurate DTCA trends and consumer exposure [37, 38]. Instead of relying on third party Marketing Firms with varying methods and estimations that appear to have variation, expansion of these transparency provisions to include DTCA disclosure would allow data to be sourced directly from manufacturers, thereby allowing for better quality data and subsequently evidence-based policymaking to curb negative impacts of pharmaceutical marketing on consumers [1]. If designed properly, this approach could identify specific pharmaceutical classes/products that are heavily targeted at consumers, types of eDTCA media/channels utilized, identification of possible illegal off-label promotion activities, and importantly, finally provide information on the geographic reach of DTCA spending outside of the USA and New Zealand [1].

### Limitations

The most significant limitation to the data we examined is that methodologies for data collection employed by different Marketing Firms may not be consistent, making it hard to assess whether data is comparable and accurate. For example, when comparing Internet DTCA Nielsen Co. expenditure data to Internet DTCA Kantar Media data, we discovered variation [20]. Reported Internet DTCA expenditure for the same 2005-2009-time period (adjusted for 2010 Consumer Price Index – All Urban), showed significant differences in expenditure amounts (p = 0.0229). We report that Kantar Media Internet DTCA expenditures’ 2005–2009 trend is not significantly different than Nielsen Co. Internet DTCA expenditures’ 2005–2009 trend (p = 0.8554), as the former’s overall increase is 72 % and the latter’s overall increase is 90 %. However, the result of this statistical test does not convey the widely fluctuating year-to-year variations in reported DTCA expenditures between these two data sources, which range from $23 million in 2007 to $191 million in 2009, with an average difference of over $100 million. In addition, methodologies for collecting eDTCA may still be in maturation and may vary based on marketing surveillance/sampling methods. As an example, in 2010 Kantar Media reported a substantial decline in Internet DTCA from $312 to 202 million (2010 Nielsen Internet DTCA data was not available for comparison). Accounting for this in the overall growth trend would mean that Internet DTCA expenditures experienced a more modest increase of 11.6 % from 2005–2010. This deduction would be a problem not only because it obscures the 72 % increases from 2005–2009, but also because this dramatic downturn makes a deviation from other data sources more likely. There are a number of reasons why reported Marketing Firm data may report variation. This includes differences in DTCA data collection methodology (i.e. media sampling and channel monitoring strategies), differences in defining the pharmaceutical market/DTCA mediums, and differences in methods for calculating expenditures. Overall inconsistency between DTCA data sources creates barriers in interpreting just how much spending is involved in pharmaceutical eDTCA marketing and makes policy analysis and public policy formulation all the more challenging.

## Conclusions

21^st^ century healthcare is increasingly becoming digital and is changing the way clinicians, patients, and the broader global public, search for and interpret information about their health status and available treatment options. Hence, the “digitization” and globalization of DTCA through forms of eDTCA, requires further study to assess its potential impact on both domestic and global healthcare outcomes, expenditures, and appropriate utilization of prescription drugs. Yet, current publicly available data on DTCA and eDTCA is extremely limited and lacks necessary validity to inform important health policy decisions. Better data is especially needed given the rise, fall, and evolution of DTCA mediums beginning with early print and radio ads, moving to national TV commercials, and now the emergence of eDTCA with the rise of the “Health Internet” and e-patient [10, 12, 39]. In response, regulatory and transparency responses such as the U.S. Sunshine Act that compels manufacturer disclosure, may provide an important basis for establishing accurate and evidence-based pharmaceutical policy that is adaptive and can dynamically adjust to the changing nature of DTCA.

### Ethics approval and consent

Not applicable.

### Standards of reporting

Not applicable.

### Data availability

We reviewed publicly available data on DTCA expenditures that is available per the references included in this study. Certain DTCA expenditure data may be subject to copyright protections limiting its public posting or availability.

## Appendix

**Appendix Table 1:** Publicly-available expenditure data used in this study, US Dollars

Category	Source	2005	2006	2007	2008	2009
Aggregate DTCA	IMS Health	$4,251,000,000	$4,898,000,000	$4,907,000,000	$4,364,000,000	$4,364,000,000^b^
	Kantar Media	$4,650,000,000	$5,410,000,000	$5,180,000,000	$4,660,000,000	$4,770,000,000
	Nielsen Co.	$4,842,992,880	$5,521,235,189	$4,804,707,359	$4,416,132,619	$4,463,925,591
	PLoS One *(Kornfield et al.)*	$5,231,000,000	$5,891,000,000	$5,483,000,000	$4,738,000,000	$4,868,000,000
Internet DTCA	Kantar Media	$181,000,000	$230,000,000	$105,000,000	$141,000,000	$312,000,000
	Nielsen Co.	$56,180,283	$92,663,966	$77,461,208	$89,733,241	$117,403,346
TV^a^	Nielsen Co.	$3,390,587,472	$3,617,308,130	$3,020,695,842	$2,959,199,600	$2,943,000,894
Print	Nielsen Co.	$1,320,786,065	$1,725,085,052	$1,663,197,318	$1,340,431,981	$1,349,517,112
Radio	Nielsen Co.	$66,798,340	$72,795,222	$36,681,209	$21,852,164	$46,323,067
Outdoor	Nielsen Co.	$8,640,720	$13,382,819	$6,671,782	$4,915,633	$7,598,381

^a^Includes network, cable, syndicated, and spot TV ads. Excludes Spanish Language TV DTCA

^b^Excludes $82,791 in reported free standing insert coupons for DTCA
